# Central Precocious Puberty in Males, GnRH Analog Treated or Untreated, Did Not Impact Fertility and Health in Midadulthood

**DOI:** 10.1210/clinem/dgaf427

**Published:** 2025-07-28

**Authors:** Liora Lazar, Tal Oron, Michal Yackobovitch-Gavan, Ariel Tenenbaum, Moshe Phillip, Joseph Meyerovitch, Liat de Vries

**Affiliations:** The Jesse Z and Sara Lea Shafer Institute for Endocrinology and Diabetes, Schneider Children's Medical Center of Israel, Petach Tikva 4920235, Israel; Gray Faculty of Medical & Health Sciences, Tel-Aviv University, Tel-Aviv 69978, Israel; The Jesse Z and Sara Lea Shafer Institute for Endocrinology and Diabetes, Schneider Children's Medical Center of Israel, Petach Tikva 4920235, Israel; Gray Faculty of Medical & Health Sciences, Tel-Aviv University, Tel-Aviv 69978, Israel; The Jesse Z and Sara Lea Shafer Institute for Endocrinology and Diabetes, Schneider Children's Medical Center of Israel, Petach Tikva 4920235, Israel; Gray Faculty of Medical & Health Sciences, Tel-Aviv University, Tel-Aviv 69978, Israel; The Jesse Z and Sara Lea Shafer Institute for Endocrinology and Diabetes, Schneider Children's Medical Center of Israel, Petach Tikva 4920235, Israel; Gray Faculty of Medical & Health Sciences, Tel-Aviv University, Tel-Aviv 69978, Israel; The Jesse Z and Sara Lea Shafer Institute for Endocrinology and Diabetes, Schneider Children's Medical Center of Israel, Petach Tikva 4920235, Israel; Gray Faculty of Medical & Health Sciences, Tel-Aviv University, Tel-Aviv 69978, Israel; The Jesse Z and Sara Lea Shafer Institute for Endocrinology and Diabetes, Schneider Children's Medical Center of Israel, Petach Tikva 4920235, Israel; Gray Faculty of Medical & Health Sciences, Tel-Aviv University, Tel-Aviv 69978, Israel; The Jesse Z and Sara Lea Shafer Institute for Endocrinology and Diabetes, Schneider Children's Medical Center of Israel, Petach Tikva 4920235, Israel; Gray Faculty of Medical & Health Sciences, Tel-Aviv University, Tel-Aviv 69978, Israel

**Keywords:** central precocious puberty, GnRH analog, male fertility

## Abstract

**Context:**

The long-term health consequences of idiopathic central precocious puberty (ICPP) in males, whether treated or untreated, are not well established.

**Objective:**

To evaluate general health and metabolic outcomes in adult men with a history of ICPP.

**Design:**

Retrospective cohort study with age, birth year, and socioeconomic status–matched controls.

**Patients:**

One hundred eighteen men aged 29 to 48 years with a history of ICPP (68 GnRH analog treated, 50 untreated) and 351 matched controls (204 matched to the treated group and 147 to the untreated group).

**Methods:**

Data were extracted from the Clalit Health Services database and included demographics, medical history, medication records, anthropometric data, and laboratory results.

**Main Outcome Measures:**

Prevalence of reproductive issues, metabolic conditions (obesity, hypertension, hyperlipidemia, diabetes), osteoporosis, malignancy, and psychopathology.

**Results:**

The median age was 40 years in the treated and 44 years in the untreated ICPP group (*P* < .001). No significant differences were observed between treated and untreated groups in the prevalence of reproductive issues (11.8% vs 18.4%, *P* = .317), metabolic disorders (35.3% vs 34.7%, *P* = .946), cardiovascular conditions (16.2% vs 14.3%, *P* = .780), bone health problems (4.4% vs 4.1%, *P* = .931), psychopathology (19.1% vs 16.3%, *P* = .698), or malignancies (1.5% vs 8.2%, *P* = .160). Reproductive and health outcomes were also comparable between ICPP patients and their respective control groups, regardless of treatment history.

**Conclusion:**

A history of ICPP in males, whether treated or untreated, does not appear to be associated with adverse long-term outcomes in reproductive, metabolic, emotional, or oncological health. Overall, adult men with prior ICPP exhibit similar health profiles to those of the general population.

Central precocious puberty (CPP) in boys is defined by the early appearance of secondary sexual characteristics before the age of 9 years, resulting from the premature activation of the hypothalamic-pituitary-gonadal axis and elevated levels of LH and FSH (1). While an underlying central nervous system pathology is identified in about 25% to 40% of boys with CPP, in more than 60% of those affected, the cause is unknown and is classified as idiopathic (2-6). The first sign of CPP in boys is testicular enlargement, followed by the development of sexual hair. Accelerated growth and advanced bone age (BA) usually appear during midpuberty, typically at pubertal stages 3 to 4 according to Tanner (7). The purpose of treating CPP is to prevent the physical and emotional impacts of premature sexual development. Since 1981, the gold standard worldwide for treating CPP in both girls and boys has been the use of GnRH analogs (GnRHa), which effectively suppress the hypothalamic-pituitary-gonadal axis by desensitizing pituitary gonadotrophs. This treatment has demonstrated both effectiveness and safety in halting gonadal activation, with the suppression reversible after discontinuation of therapy (8, 9). However, concerns have been raised regarding the long-term effects of CPP (10, 11) and the potential side effects of GnRHa treatment. Reviews of the literature over the past 2 decades on the short- and long-term outcomes of CPP and GnRHa treatment—particularly in relation to obesity, bone health, cancer rates, metabolic comorbidities, cardiovascular risks, and reproductive function and fertility—have generally provided reassuring results (7, 12, 13). However, most long-term outcome studies on idiopathic CPP (ICPP) and GnRHa treatment have focused on women (12, 13), and very limited data are available on men with a history of ICPP (14-16). This is likely due to the substantially lower incidence, by roughly 10 times, of ICPP in boys than in girls, as was demonstrated in a population-based cohort study using the Danish National Patient Registry (17, 18). The mean annual incidence rates of ICPP reported from 1998 to 2017 were 0.9 per 10 000 boys and 9.2 per 10 000 girls.

In this study, we examined the long-term outcomes of ICPP and GnRHa treatment in men who were in the third, fourth, and fifth decades of their lives. These men were diagnosed with ICPP in childhood; some of them received GnRHa treatment. All the patients were followed at our institution until they reached full puberty. The primary objective of our study was to evaluate the general health, metabolic outcomes, and fertility of men with a history of ICPP, compared to age-matched men with normal puberty. The secondary objective was to assess whether pubertal suppressive therapy significantly impacted long-term outcomes by comparing men with a history of ICPP who had been and had not been treated with GnRHa.

## Methods

### Data Source

A retrospective cohort study with a matched control group was conducted using the electronic database of Clalit Health Services (CHS), which serves about 54% of the population in Israel. The database is a comprehensive centralized data warehouse that aggregates continuous real-time clinical and administrative input from health service providers, physicians, laboratories, and pharmacies and can be queried down to the level of an individual member. It includes patient demographics, socioeconomic status by residence [socioeconomic position (SEP), as detailed later], clinical characteristics, diagnoses from hospital discharge and outpatient clinic visits, laboratory test results, medical treatments, and medication dispensing information. Data for this study were extracted from the database using the CHS sharing platform powered by MDClone (https://www.mdclone.com).

### Ethical Approval

The study was approved by the Rabin Medical Center institutional ethics committee (approval no. 4379), and the need for consent was waived due to the retrospective retrieval of anonymized data.

### Patients

The medical files were reviewed of boys who had sought medical attention due to ICPP at the Institute for Pediatric Endocrinology at Schneider Children's Medical Center of Israel during 1980 to 2000. We identified 118 boys who fulfilled the criteria for diagnosis, treatment, and follow-up of CPP and who were currently insured by CHS.

The diagnosis of ICPP requires the onset of secondary pubertal signs (testicular volume ≥4 mL and genitalia ≥ Tanner stage 2, with or without sexual hair) before the chronological age (CA) of 9 years. The diagnosis also requires a GnRH-stimulated peak LH level greater than 5 IU/L and a normal brain magnetic resonance imaging with a thorough examination of the hypothalamic-pituitary region at diagnosis.

In accordance with our departmental policy during 1980 to 2000, pubertal-suppressive therapy was offered to all the boys diagnosed with progressive ICPP following an observation period of up to 6 months. This was intended to exclude nonsustained or slowly progressive forms of CPP. Therapy was accepted, with parental consent, by 68 boys and refused by 50. During the study period, the therapy consisted of a depot preparation of GnRHa (Decapeptyl; Ferring Pharmaceuticals Ltd., Malmo, Sweden) administered by an intramuscular injection every 4 weeks at a calculated dose of 1.5-3.0 μg/kg release per day, to a maximal dose of 3.75 mg. Treatment was generally discontinued at an age when normal puberty could be expected (at CA 12-12.5 years and BA 13-13.5 years). All the boys, treated and untreated, were regularly followed to the completion of puberty. Excluded from the study were boys born small for gestational age or with chronic disease, bone dysplasia, organic brain disease, congenital adrenal hyperplasia, or other endocrinological abnormalities. Also excluded were boys who had undergone radiation therapy or chemotherapy, who had been treated for less than 2 years, or who were noncompliant.

The control group was randomly selected at a ratio of 1:3 from males insured in CHS. To form the matched control groups, we used the MatchIt package in R, applying nearest neighbor propensity score matching. Matching was constrained to ensure exact matches on socioeconomic group. Hence treated and control individuals were only compared within the same socioeconomic group category. Year of birth was either included as a continuous covariate for approximate matching or, in sensitivity analyses, used for exact matching to further align age distributions. We used a 1:3 matching ratio without replacement, and balance was assessed postmatching to ensure comparability between groups. This approach minimized confounding variables and approximated a randomized comparison across key background characteristics.

According to the size of the cohort, the control group comprised 351 men: 204 for the GnRHa-treated group and 147 for the untreated group.

Medical information for the 118 eligible men with a history of ICPP was retrieved from the CHS computerized data warehouse using its unique personal identification numbers. All the data collected from CHS, including information for our study group and the control group, were anonymized.

### Data Retrieval

#### Data collection of the ICPP group through childhood and adolescence

Extracted from the medical files of all the boys with ICPP were CA, BA, height, body mass index (BMI), and the pubertal stage at the first visit and CA, height, and BMI at the last visit (Tanner stage 5). For the boys who were treated with GnRHa, the data obtained from the files included CA at the initiation and cessation of pubertal suppressive therapy and the duration of the treatment.

#### Data collection of the ICPP and control groups through adulthood from the CHS database

The computerized data of the studied and control groups were reviewed to provide dates of birth; most recent documentations of anthropometric measures (height, weight, or BMI); and diagnoses of chronic diseases, laboratory results, and dispensed medications.

Extracted also were the current SEP of each patient. The SEP index of the Israel Central Bureau of Statistics is an adjusted calculation of 14 variables that measures social and economic levels in the domains of demographics, education, standard of living, and employment. SEP by home address was based on the 2021 classification of towns and neighborhoods of cities (19). SEP clusters are ranked 1 to 10, with 1 representing the lowest SEP. For this study, the 10 clusters were categorized to low SEP (clusters 1-4), medium SEP (clusters 5-6), and high SEP (clusters 7-10).

The following medical issues were assessed, with their variables analyzed and compared across groups, both as specific components of each issue and as a combined measure of all the components.

##### Reproductive issues

These were identified based on documented visits to fertility clinics and included erectile dysfunction, infertility diagnosis in medical records, and recorded purchases of medications specifically prescribed for infertility treatment.

##### Obesity

This was defined according to World Health Organization criteria: normal weight, BMI <24.9 kg/m^2^; overweight, BMI 25.0-29.9 kg/m^2^; adiposity grade I, BMI 30.0-34.9 kg/m^2^; adiposity grade II, BMI 35.0-39.9 kg/m^2^; adiposity grade III, BMI >40 kg/m^2^.

##### Evidence of metabolic syndrome

This was identified according to data on diabetes mellitus, dyslipidaemia, or hypertension. Type 2 diabetes mellitus was considered a diagnosis if it was recorded in the CHS database or there was a record of diabetes medications or documented hemoglobin A1c (HbA1c) ≥ 6.5%. Dyslipidemia was considered a diagnosis if hyperlipidemia was recorded in the CHS database or there was a record of prescriptions for lipid-lowering medications. Hypertension was considered a diagnosis if hypertension was recorded in the CHS database or there was a record of antihypertensive medications.

##### Cardiovascular disease

This was considered a documented diagnosis if 1 or more of the following conditions were met: ischemic heart disease, myocardial infarction, transient ischemic attack, or cerebrovascular accident (stroke). Also considered were documented cardiac procedures (percutaneous transluminal coronary angioplasty or coronary artery bypass graft) and consultations with cardiologists and documented visits to a cardiology clinic.

##### Bone health

Bone health, particularly related to reduced bone density, was considered a documented diagnosis in the presence of osteopenia or osteoporosis, a record of medications used for bone health including bisphosphonates, and reports of calcium and vitamin D supplements.

##### Malignancy

This was considered a documented diagnosis if malignant diseases, visits to oncology clinics, or a record of chemotherapy or radiation therapy were present.

##### Emotional issues

Emotional issues including depression, anxiety, and personality disorders were considered documented diagnoses in the presence of visits to mental health clinics or recorded purchases of medications for mental health conditions.

##### Laboratory test results

These included values for lipids (total cholesterol, low-density lipoprotein cholesterol, high-density lipoprotein cholesterol, and triglycerides), glucose levels, HbA1c, hormones (LH, FSH, and testosterone), and prostatic-specific antigen. In line with CHS policy, all blood samples were collected between 7:30 and 9:30 in the morning, ensuring consistency across participants.

### Statistical Analysis

The data were analyzed using IBM SPSS Statistics for Windows v29 (IBM Corp., Armonk, NY). The data are presented as mean ± SD for variables with a normal distribution, median and interquartile range for variables with a skewed distribution, and n (percent) for categorical variables.

Between-group comparisons were evaluated using an independent samples *t*-test for variables with a normal distribution, the Mann-Whitney U-test for variables with a skewed distribution, and Pearson's chi-square test for categorical variables.

To compare health status and metabolic outcomes between men with a history of ICPP, treated and untreated with GnRHa, we adjusted all analyses for age due to a significant difference in age at the time of data collection. Logistic regression was used for all dichotomous variables, linear regression for BMI as a continuous variable, and ordinal regression for BMI as an ordinal variable.

## Results

The study group comprised 118 men diagnosed with ICPP, aged 29 to 48 years. Of them, 68 had been treated with GnRHa and 50 had not. The median age (interquartile range) at the time of data collection was 40 (29, 44) years among men treated with GnRHa and 44 (40, 46) years among those not treated (*P* < .001). The control group included 351 men matched for age and SEP, selected based on their year of birth and community clinic affiliation. Of them, 204 were matched with the GnRHa-treated group and 147 with the untreated group.

### Characteristics of the ICPP Group, From Diagnosis to Late Adolescence

The characteristics of the boys with ICPP at diagnosis and at their final follow-up visit in our clinic are summarized in Table 1. The median age at puberty onset, anthropometric data, and pubertal stage at presentation were similar between those who had and had not been treated with GnRHa. Similarly, at the final follow-up visit, anthropometric measurements remained comparable between the 2 groups. BMI SD score was within the normal range at diagnosis and remained stable at the final visit.

**Table 1.** Clinical and anthropometric characteristics of boys with ICPP: comparison between GnRHa-treated and -untreated participants

	Treated with GnRHa n = 68	Not treated with GnRHa n = 50	*P*
First visit			
CA, median (IQR), yrs	9.0 (8.4, 9.5)	9.1 (8.5, 9.6)	.447
BA SDS, median (IQR)	1.61 (1.15, 2.38)	1.34 (0.72, 1.84)	.018
Ht SDS, mean ± SD	0.27 ± 1.08	−0.06 ± 1.05	.101
BMI SDS, median (IQR)	0.15 (−0.35, 0.50)	0.09 (−0.66, 0.70)	.944
Pubertal stage (Tanner), n (%)			
2	40 (58.8)	21 (42.0)	.071
3	28 (41.2)	29 (58.0)	
GnRHa treatment			
CA at initiation, median (IQR), yrs	10.3 (9.6, 10.8)		
CA at discontinuation, median (IQR), yrs	13.3 (12.6, 13.8)		
Duration, yrs	3 (2.6, 3.4)		
Last visit			
CA, median (IQR), yrs	17.0 (16.2, 17.6)	16.0 (15.5, 16.5)	<.001
Ht, median (IQR), cm	167.6 (164.3, 173.7)	166.0 (163.1, 169.3)	.088
Ht SDS, mean ± SD	−0.785 ± 0.918	−0.798 ± 0.966	.983
BMI, median (IQR), kg/m^2^	21.5 (20.6, 22.6)	20.8 (18.5, 22.3)	.029
BMI SDS, median (IQR)	0.150 (−0.21, 0.60)	0.110 (−0.87, 0.59)	.334
MPHt, median (IQR), cm	171.3 (166.8, 174.6)	168.6 (164.9, 173.0)	.097
Ht-MPHt difference, median (IQR), cm	−2.1 (−5.8,2.5)	−3.5 (−6.6, 0.83)	.192

The data are presented as mean ± SD for variables with a normal distribution, median and IQR for variables with a skewed distribution, and n (%) for categorical variables.

*P*-values represent between-group differences using the independent samples *t*-test for variables with a normal distribution, Mann-Whitney U-test for variables with a skewed distribution, and Pearson’s chi-square tests for categorical variables.

Abbreviations: BA, bone age; BMI, body mass index; CA, chronologic age; GnRHa, GnRH analog; Ht, height; IQR, interquartile range; MPHt, midparental height; SDS, SD score; yrs, years.

In both groups, the achieved height was comparable to the midparental height.

### Characteristics of the ICPP Group in Adulthood

#### SEP

Among those with a history of ICPP, the SEP index was similar between those who had and had not been treated with GnRHa. Among those who had been treated with GnRHa, SEP was classified as low in 6 (9.0%), medium in 10 (14.9%), and high in 51 (76.1%). Among those who had not been treated, SEP was low in 4 (8.5%), medium in 9 (19.1%), and high in 34 (72.3%) (*P* = .837). In accordance with the procedure determined for selecting the control groups, their SEP status was matched to that of the study groups.

The clinical and laboratory indices of the men with a history of ICPP and the men matched as controls are detailed in Tables 2 and 3.

**Table 2.** Health status and metabolic outcomes in men with a history of ICPP: comparison between GnRHa-treated and -untreated patients and their matched controls

	ICPP, treated	Controls	*P*_1_ *^a^*	ICPP, untreated	Controls	*P*_2_ *^b^*	*P*_3_ *^c^*
Number at data collection	68	204		50	147		
Age, median (IQR), yrs	40 (29, 44)	40 (29, 44)	1.00	44 (40, 46)	44 (40, 46)	1.00	<.001
Age range, yrs	22-52	22-52		26-54	26-54		
Reproductive issues, n (%)	8 (11.8)	33 (16.2)	.379	9 (18.4)	39 (26.5)	.250	.840
Fertility clinic visits	7 (10.3)	23 (11.3)	.823	6 (12.2)	32 (21.8)	.144	.749
Infertility diagnosis	2 (2.9)	2 (1.0)	.261	2 (4.1)	4 (2.7)	.641	.776
Infertility Rx	0	3 (1.5)	.576	3 (6.1)	2 (1.4)	.101	.997
Erectile dysfunction	1 (1.5)	11 (5.4)	.305	4 (8.2)	8 (5.4)	.491	.392
Erectile dysfunction age	33.3	33.5 ± 4.0		45.9 ± 1.3	40.4 ± 3.2	.009	
Alopecia, n (%)	2 (2.9)	7 (3.4)	.845	2 (4.1)	7 (4.8)	.884	.731
Acne, n (%)	2 (2.9)	11 (5.4)	.528	5 (10.2)	7 (4.8)	.179	.111
BMI, median (IQR), kg/m^2^	25.7 (22.6, 29.8)	25.1 (22.4, 28.1)	.366	24.9 (22.7, 28.1)	24.9 (22.8, 27.7)	.751	.947
BMI distribution, n (%), kg/m^2^							
<18.5	2 (4.3)	3 (1.6)		1 (2.8)	2 (1.4)		
18.5-24.9	21 (44.7)	77 (40.1)		11 (30.6)	56 (39.2)		
25-29.9	14 (29.8)	75 (39.1)	.398	18 (56.3)	56 (39.2)	.718	.837
30-34.9	7 (14.9)	32 (16.7)		4 (11.1)	22 (15.4)		
>35	3 (6.4)	5 (2.6)		2 (5.6)	7 (4.9)		
Metabolic disorders, n (%)	24 (35.3)	73 (35.8)	.942	17 (34.7)	67 (45.6)	.182	.261
Obesity rate	13 (19.1)	38 (18.6)	.929	7 (14.3)	35 (23.8)	.159	
Antiobesity Rx	1 (1.5)	2 (1.0)	.737	2 (4.1)	3 (2.0)	.600	
Type 2 diabetes	3 (4.4)	6 (2.9)	.695	1 (2.0)	6 (4.1)	.683	
Hyperlipidemia	16 (23.5)	54 (26.5)	.631	11 (22.4)	43 (29.3)	.356	
Lipid-lowering Rx	8 (11.8)	17 (8.3)	.396	4 (8.2)	13 (8.8)	.884	
Sleep disorders	6 (8.8)	3 (1.5)	.009	5 (10.2)	4 (2.7)	.045	.826
Cardiovascular issues, n (%)	11 (16.2)	39 (19.1)	.588	7 (14.3)	26 (17.7)	.582	.328
Hypertension	2 (2.9)	18 (8.8)	.108	3 (6.1	15 (10.2)	.570	.503
Cardiology clinic visits	10 (14.7)	39 (19.1)	.412	7 (14.3)	26 (17.7)	.582	.491
CVD disease diagnosis	1 (1.5)	2 (1.0)	.737	0	2 (1.4)	.412	
Cardiac procedures	2 (2.9)	10 (4.9)	.736	2 (4.1)	10 (6.8)	.734	.861
Bone health problems, n (%)	3 (4.4)	2 (1.0)	.102	2 (4.1)	1 (0.7)	.155	.786
Osteoporosis diagnosis	3 (4.4)	2 (1.0)	.102	2 (4.1)	1 (0.7)	.155	.786
Osteoporosis age range, years	22.9-37.8	29.9, 46.5		26.0, 31.8	36.9		
Issued medications	10 (14.7)	15 (7.4)	.069	4 (8.2)	14 (9.5)	.755	.067
Malignancy, n (%)	1 (1.5)	9 (4.4)	.460	4 (8.2)	4 (2.7)	.109	.177
Emotional problems, n (%)	13 (19.1)	27 (13.2)	.236	8 (16.3)	25 (17.0)	.912	.715
Depression	13 (19.1)	19 (9.3)	.03	6 (12.0)	18 (12.2)	.999	.085
Personality disorders	1 (1.5)	1 (0.5)	.438	1 (2.0)	2 (1.4)	.737	.686
Anxiety	2 (2.9)	14 (6.9)	.372	2 (4.1)	13 (8.8)	.365	.943

The data are presented as median and IQR for variables with a skewed distribution and as n (%) for categorical variables.

*P*-values represent between-group differences using the independent samples *t*-test for variables with a normal distribution, Mann-Whitney U-test for variables with a skewed distribution, and Pearson’s chi-square tests for categorical variables.

Abbreviations: BMI, body mass index; CVD, cardiovascular disease; GnRHa, GnRH analog; ICPP, idiopathic central precocious puberty; IQR, interquartile range; Rx, prescription; yrs, years.

^*a*^Comparisons between GnRHa-treated men with a history of ICPP and their matched controls.

^*b*^Comparisons between untreated men with a history of ICPP and their matched controls.

^*c*^Comparisons between GnRHa-treated and -untreated men with a history of ICPP, adjusted for age using regression models.

**Table 3.** Laboratory findings of in men with a history of ICPP: comparison between GnRHa-treated and -untreated patients and their matched controls

	ICPP, treated	Controls	*P*_1_ *^a^*	ICPP, untreated	Controls	*P*_2_ *^b^*	*P*_3_ *^c^*
Total number	68	204		50	147		
gonadotropins							
Number tested	7	15		6	16		
Age at testing, mean ± SD, yrs	31.0 ± 9.4	37.5 ± 4.6	.120	39.3 ± 5.6	33.8 ± 6.3	.074	.078
LH, median (IQR), IU/L	3.1 (2.9, 4.7)	3.6 (3.0, 4.9)	.535	3.9 (3.3, 5.6)	3.9 (2.8, 5.6)	.747	.534
FSH, median (IQR), IU/L	3.5 (1.8, 10.9)	3.9 (2.9, 6.8)	.913	7.0 (5.1, 15.3)	4.7 (2.7, 5.6)	.068	.092
Testosterone							
Number tested	8	23		9	26		
Age at testing, mean ± SD, yrs	34.2 ± 7.4	37.4 ± 5.5	.202	39.5 ± 7.8	36.8 ± 6/3	.299	.172
Testosterone, mean ± SD, nmol/L	15.8 ± 7.7	12.3 ± 6.8	.266	14.1 ± 6.4	13.6 ± 6.5	.856	.617
PSA							
Number tested	8	25		9	27		
Age at testing, mean ± SD, yrs	37.1 ± 7.5	39.6 ± 8.2	0.44	40.7 ± 5.8	41.3 ± 7.9	.842	.277
PSA, median (IQR), μg/L	0.48 (0.38, 0.72)	0.66 (0.43, 0.87)	.290	0.90 (0.40, 1.32)	0.50 (0.38, 0.88)	.233	.139
Lipids							
Number tested	50	180		37	133		
Total cholesterol, median (IQR), mg/dL	170 (140, 188)	177 (152, 207)	.054	177 (154, 192)	184 (161, 212)	.198	.180
HDL cholesterol, median (IQR), mg/dL	44 (39, 55)	46 (40, 54)	.251	46 (38, 52)	46 (40, 52)	.734	.834
LDL cholesterol, median (IQR), mg/dL	100 (76, 122)	103 (87, 127)	.141	101 (88, 122)	111 (95, 132)	.856	.617
Triglycerides, median (IQR), mg/dL	101 (78, 160)	112 (71, 166)	.794	125 (73, 195)	117 (84, 167)	.299	.172
Glucose metabolism							
Number tested	53	184		39	136		
Glucose, median (IQR), mg/dL	87 (81, 96)	88 (82, 94)	.978	90 (83, 99)	90 (84, 99)	.233	.139
HbA1c, median (IQR), %	5.2 (4.9, 5.5)	5.2 (5.0, 5.5)	.641	5.4 (5.2, 5.6)	5.2 (4.8, 5.4)	.842	.277

The data are presented as mean ± SD for variables with a normal distribution and as median and IQR for variables with a skewed distribution.

*P*-values represent between-group differences using the independent samples *t*-test for variables with a normal distribution, Mann-Whitney U-test for variables with a skewed distribution, and Pearson’s chi-square tests for categorical variables.

Abbreviations: GnRHa, GnRH analog; HbA1c, hemoglobin A1c; HDL, high-density lipoprotein; IQR, interquartile range; LDL, low-density lipoprotein; PSA, prostate-specific antigen; yrs, years.

^*a*^Comparisons between GnRHA-treated men with a history of ICPP and their matched controls.

^*b*^Comparisons between untreated men with a history of ICPP and their matched controls.

^*c*^Comparisons between GnRHa-treated and -untreated men with a history of ICPP.

#### Reproductive outcomes

Among men with a history of ICPP, the incidence of reproductive issues was similar between those who had and had not been treated with GnRHa (11.8% vs 18.4%, *P* = .317). These incidences were similar to those of their matched controls (treated ICPP 11.8% vs 16.2%, *P* = .379 and untreated ICPP 18.4% vs 26.5%, *P* = .250, respectively). This trend persisted in analyses of individual components of reproductive outcomes, including fertility clinic visits, infertility diagnosis, and erectile dysfunction.

In a subset of the patients with data on serum levels of LH, FSH, testosterone, and prostatic-specific antigen, the results fell within the normal range for adult males. No significant differences were noted between the study and control groups.

Neither the prevalence of acne nor alopecia differed among those with ICPP between those who had and had not been treated with GnRHa or between each of these groups and their matched controls.

#### Obesity and obesity-related complications

At the time of data collection, no significant differences were identified in median BMI, obesity rates, or BMI distribution among those with ICPP between those who had and had not been treated with GnRHa or between each of these groups and their matched controls.

There were no significant differences in the prevalence of sleep disorders (including obstructive sleep apnea) between individuals with ICPP who were treated with GnRHa and those who were not or between either of these groups and their matched controls.

#### Metabolic complications

The rates of type 2 diabetes and dyslipidaemia requiring lipid-lowering medications were all below 10% and were similar between the study groups and between each study group and its control group. Mean fasting glucose, HbA1c, total cholesterol, low-density lipoprotein cholesterol, high-density lipoprotein cholesterol, and triglyceride levels did not differ significantly between the study groups or between each study and its matched control group.

#### Cardiovascular diseases

Among the men with ICPP, the prevalence of cardiovascular diseases, including both suspected and confirmed diagnoses, was 16.2% among those who had been treated with GnRHa and 14.3% among those who had not been treated. For their respective control groups, the prevalences were 19.1% and 17.7%. All the differences between the study and control groups were nonsignificant (*P* > .05). The prevalence of each diagnosis, including hypertension, confirmed cardiovascular disease, and the documentation of cardiac procedures, was low across all groups.

#### Bone health disorders

The prevalence of osteopenia and osteoporosis and of medications issued for bone health was low in both study groups and did not differ significantly from their control groups.

#### Malignancy

The malignancy rate was below 10% among the men with a history of ICPP, whether treated or untreated with GnRHa. Statistically significant differences were not observed between the study groups and their respective control groups.

#### Mental health

The rate of documented emotional problems was under 20%. Among those with a history of ICPP, significant differences were not observed between those treated and not treated with GnRHa or between the study groups and their matched controls.

## Discussion

In the present study, we found that the overall health of men with a history of ICPP was similar to that of men who underwent normal puberty. ICPP, whether treated or untreated with GnRHa, was not associated with a higher prevalence of overweight, obesity, metabolic disorders, or cardiovascular complications. Furthermore, there was no increased risk of bone health issues, cancer, or emotional disorders. Additionally, neither ICPP nor GnRHa treatment appeared to have any long-term impact on future reproductive function.

While preserving growth potential is 1 of the reasons for initiating GnRHa therapy, a major consideration is the prevention or mitigation of psychological distress and social challenges resulting from the hormonal and physical changes of premature puberty. Although our data did not reveal a significant difference in adult height between treated and untreated individuals, this does not diminish the overall therapeutic value of the treatment, including its favorable long-term safety profile.

It is well established that ICPP does not affect reproductive function (20). However, despite GnRHa being the primary treatment for ICPP since the early 1980s (21, 22), knowledge of the long-term impact of GnRHa on gonadal function in men with a history of ICPP is still limited.

Previous studies reported normal function of the pituitary-testicular axis with sex hormone levels and sperm analysis shortly after the cessation of GnRHa therapy. This was consistent with adult values in all the boys treated with GnRHa for CPP (15, 16). However, those studies were conducted during late adolescence and involved relatively small numbers of individuals, limiting the available information on fertility and paternity outcomes in adulthood. Our results of men in their third through fifth decades of life are promising. Adult men with a history of ICPP, regardless of treatment with GnRHa, experienced a low incidence of reproductive issues and sexual dysfunction, similar to those of matched controls. Several of our patients lacked data on hormone levels, sperm analysis, and paternity information. Nonetheless, we believe that our collection of data on fertility-related diagnoses, referrals to specialists, visits to male fertility clinics, and prescribed fertility medications provides reliable assessments of fertility issues in both the patient and control groups. Although erectile dysfunction is not a cause of infertility in the biological sense, it can pose significant reproductive challenges. Erectile dysfunction may result from a range of factors, including psychogenic factors, medications, substance or alcohol abuse, hormonal disturbances, cardiovascular or metabolic conditions, and neurological disorders. Given the relatively young age and overall favorable metabolic and cardiovascular profiles of our cohort, it is less likely that erectile dysfunction in this group is associated with cardiovascular or metabolic disorders.

Early onset of puberty and GnRHa treatment have been linked to increased adult adiposity, particularly in women (11). However, the data on the natural progression, from late adolescence to young adulthood, of weight gain in males with a history of ICPP and of the prevalence of obesity and its related comorbidities remain scarce and inconsistent (23). Among boys diagnosed with ICPP, both treated and not treated with GnRHa, we report a BMI SD score within the normal range, which remained stable at the final visit. Our results are consistent with our previous findings in women with a history of ICPP, which showed similar weight status to that of the general population (13). Here, among men with a history of ICPP, in their third to fifth decades of life, both treated and untreated with GnRHa, the BMI distribution was comparable to that of the general population. Rates of overweight or obesity were not higher. These findings align with those of Palmert et al (24), who reported that while 71% of boys with CPP had a BMI above the 85th percentile before starting GnRHa treatment, only 50% of those treated with GnRHa had a BMI above the 85th percentile at the end of therapy. This suggests that GnRHa treatment does not significantly impact weight gain in boys with CPP. Leite et al (25) found that boys experienced a reduction in BMI during GnRHa treatment and that this effect lasted for 1 year after termination of treatment.

The long-term impact of sexual precocity on the general health status of men with a history of ICPP remains inconclusive, especially in relation to metabolic issues, cardiovascular diseases, and malignancy (11). Women with CPP or early menarche who developed obesity in adulthood were shown to be more likely to develop metabolic issues (such as dyslipidemia and diabetes), hypertension, and cardiovascular disease (11, 26-28). These negative health outcomes were linked to excessive adult adiposity and hyperinsulinism. The BMI of our male patients with ICPP was not elevated during childhood and adolescence and remained within the normal range for the majority of the study cohort. Therefore, it is not surprising that the incidences of obesity-related metabolic and cardiovascular comorbidities and hypertension were relatively low and similar to those of the age-matched controls. The actual rates of confirmed hypertension and cardiovascular disease diagnoses were even lower, as several of those with suspected diagnoses, which prompted referral to the cardiology clinic, were ultimately ruled out. Malignancy was recorded in only 5 of the men with a history of ICPP. The incidence was very low, both among those treated and not treated with GnRHa, and not significantly different from that of their controls. While this result is reassuring, the lower malignancy rate may be partially due to the relatively young age of the patients at the time of data collection. Therefore, additional long-term follow-up is needed. Bone mineral density (BMD) is another key factor to consider in the long-term follow-up of those treated with GnRHa. In adults, GnRHa therapy induces a hypogonadal state, which may lead to decreased BMD and an increased risk of fractures (29). Concerns have been raised that by slowing bone mineral accrual, prolonged suppression of the pituitary-gonadal axis with GnRHa during puberty may impair the development of peak bone mass (30). However, the report of normal lumbar area and volumetric BMD in a small group of men treated with GnRHa (20) suggests that there may be no long-term negative impact on peak bone mass. It seems that after discontinuation of therapy, bone accrual resumes, and BMD appears to normalize by late adolescence (15, 20). Our findings are consistent with those observations. Specifically, the proportions with osteoporosis or using osteoporosis medications were similar between those who had and had not been treated with GnRHa during puberty and to the control groups without ICPP.

Psychological distress, including feelings of isolation or insecurity, is common in children with CPP. Boys with early-onset puberty can have behavioral difficulties and poor psychological adjustment (31). Girls may experience increased stress from early breast development and menses. Furthermore, girls who experience early menarche are also at risk of depressive symptoms and antisocial behaviors during adolescence and continuing into early-middle adulthood; they may also have a lower quality of life (32, 33). While many formerly treated individuals adapt well over time, emotional difficulties may continue into adulthood. Our findings of similar rates of emotional issues among men with a history of ICPP compared to their peers without ICPP are reassuring.

### Strengths and Limitations

A key strength of our study is the analysis of data from a large cohort of men with a history of ICPP who were and were not treated with GnRHa. They were in early and midadulthood, spanning 1 to 3 decades after their last clinic visit. Matched control groups were drawn from a comprehensive health information system. By extracting a wide range of socioeconomic, anthropometric, clinical, and laboratory data from both outpatient and inpatient settings, we were able to thoroughly evaluate the impact of ICPP and its treatment in individuals aged 29 to 48 years.

However, this study has several limitations. Its retrospective design may have led to missing or incomplete data, particularly regarding hormonal and fertility measures, limiting conclusions about long-term hormonal effects. The relatively small sample size and young median age may underestimate risks that emerge later in life, such as cancer or osteoporosis.

The cohort included only males with ICPP referred to our endocrine clinic, a group generally of higher socioeconomic status than the broader Israeli population, possibly reflecting increased parental concern about puberty and growth. Participants were also all insured by CHS, Israel's largest public healthcare provider, which covers about 54% of the population. While this offers broad representation, findings may not be fully generalizable to all individuals with ICPP. Yet, Israel's public healthcare system ensures equal access across all 4 national insurance providers. Lastly, we could not account for certain lifestyle and familial factors—such as physical activity, smoking, diet, and family history of chronic disease—which may influence the outcomes assessed.

In conclusion, our study demonstrated similar general health status in early to midadulthood, of men with a history of ICPP, whether treated with GnRHa or not, to that of the general population. These findings contribute to the growing body of evidence supporting the long-term safety of GnRHa treatment for ICPP, thus reassuring clinicians and families. The comparable health outcomes among men, between those treated and not treated with GnRHa, suggest that treatment decisions can be based on individual clinical factors, without substantial concern regarding long-term adverse effects.

## Data Availability

Anonymized participant data and study documents can be made available by the corresponding author upon reasonable request.
