# Correction to: “Central Precocious Puberty in Males, GnRH Analog Treated or Untreated, Did Not Impact Fertility and Health in Midadulthood”

**DOI:** 10.1210/clinem/dgaf508

**Published:** 2025-09-16

In the above-named article by Lazar L, Oron T, Yackobovitch-Gavan M, Tenenbaum A, Phillip M, Meyerovitch J, and de Vries L (*J Clin Endocrinol Metab*. 2025; doi: 10.1210/clinem/dgaf427) there was an error in the Table 2.

In the originally published article, in Table 2, under the fifth column titled “Controls,” in the second row titled “Age, median (IQR), yrs,” the median age for controls was written as “4 (40, 46).” In the corrected article, “4 (40, 46)” has been corrected to read “44 (40, 46).”

The article has been corrected online.

doi: 10.1210/clinem/dgaf427

